# Extra short 4mm implants used to rehabilitation of atrophic posterior mandible. A serial case reports

**DOI:** 10.4317/jced.56654

**Published:** 2020-05-01

**Authors:** Alysson-Henrique-Neves Ramos, Gianfilippo-Machado Cornacchia, Eduardo Nunes, Mauricio-Greco Cosso, Leandro-Napier de Souza, Elton-Gonçalves Zenóbio

**Affiliations:** 1 Postgraduate Program in Dentistry, Pontifical Catholic University of Minas Gerais, Belo Horizonte, Minas Gerais, Brazil; 2 Adjunct Professor IV, Department of Dentistry, Pontifical Catholic University of Minas Gerais, Belo Horizonte, Brazil; 3 Adjunct Professor, Department of Oral Surgery and Pathology, School of Dentistry, Universidade Federal de Minas Gerais

## Abstract

Rehabilitation of patients through implants in areas with severe bone resorption in the posterior mandible is a challenge in implant dentistry. In this context, extra short implants configure a treatment option for this type of patient, as they can avoid increased financial cost, treatment time and patient morbidity. The present study evaluated the marginal bone stability in individualized extra-short implants for masticatory function in the posterior mandible. Using digitized periapical radiographs of 13 extra-short implants performed on 7 patients. The mesial and distal regions of each implant were selected, from the bone crest to the region parallel to the apex, and the bone stability of this crest was measured using the Image J software immediate T1 and 1 year after rehabilitation (T2). The height of the bone crest remained stable, showing no statistically significant difference between T1 and T2 (p> 0.005) for both the mesial bone crest and the distal bone crest in individual or united crowns rehabilitation. Marginal bone stability was observed in extra short implants, corroborating the biological and biomechanical stability of these implants presented in the literature. Despite the limited sample size and proservation time, extra-short implants are predictive treatment options for patients with severe bone atrophy in the posterior mandible.

** Key words:**Extra-short implants, marginal bone loss, mandibular bone atrophy.

## Introduction

Individuals with posterior mandibular bone atrophy who require implant rehabilitation may need more invasive surgeries to reconstruct bone tissue and subsequent fixation of regular implants. Bone regeneration surgeries, such as osteogenic distraction, block bone grafts and alveolar nerve lateralization are indicated (1-4). These surgical procedures provide increased in treatment time, morbidity and higher cost to the patients (5). In this specific clinical situation, extra short implants are indicated, being a viable alternative treatment (6).

Studies that provide an objective classification for extra short and short implants could be used as the study (7), that in a retrospective study between 2001 and 2009, in which 128 implants were used, classified the implants into extra short and short with measurements between 5.5 to 8.5mm. However, after a retrospective study of scientific articles from January 2004 to February 2016, with keywords “dental implant length and dental implant diameter” classified the extra-short implants to be less than or equal to 6mm, shorter implants with more than 6mm and less than 10mm, and regular greater than or equal to 10mm and less than 13mm and finally longs that are greater than or equal to 13mm (8).

Short implants present a simple surgical technique compared to bone reconstruction surgeries used to fix standard implants, reducing treatment time and morbidity (9).

Biomechanical aspects and marginal bone loss of short implants compared to regular sized implants, studies show that there was no significant difference over a period of 2 to 3 years. However, long-term clinical studies are lacking (10).

The survival of short and extra-short implants is independent of implant diameter and implant crown ratio, as well as macrogeometry and prosthesis type (11).

Extra-short implants (4 mm in length) are presented as a treatment option in patients with severe jaw resorption (12). In their case study, 10 patients were selected. Each patient received 6 mandible implants, 2 anterior implants (inter-foramen) 10mm high and 4 additional 4 mm height implants in the posterior region distributed bilaterally. They also found that stability and marginal bone loss were similar in both implant sizes.In the context of evaluating the predictability of extra-short implants, according to the classification (8), the present study evaluated marginal bone stability in extra-short implants for more than one year of single or united rehabilitation in serial cases.

## Case Report

Seven patients from the University Dentistry Department aged 55 to 78 years, both gender, non-smokers, without systemic diseases were selected. The patients showed mandibular posterior bone atrophy, characterized by available bone height less than or equal to 7mm measured from the upper cortical of the canal of the inferior alveolar nerve plexus. These patients were rehabilitated with 13 extra-short implants, properly following all commercial system surgical protocols for such procedure.

The implants used were Straumann implants, Tissue Level model with measures of 4 mm in height and variable width of 4.1 mm or 4.8 mm.

**Figure 1:**
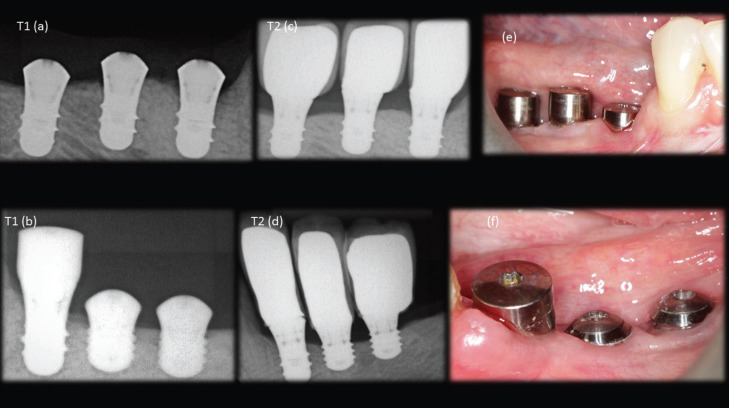
a,b) immediate x-ray of the implants installed; c,d) radiograph after one year of masticatory function; e,f) clinical photography.

**Figure 2:**
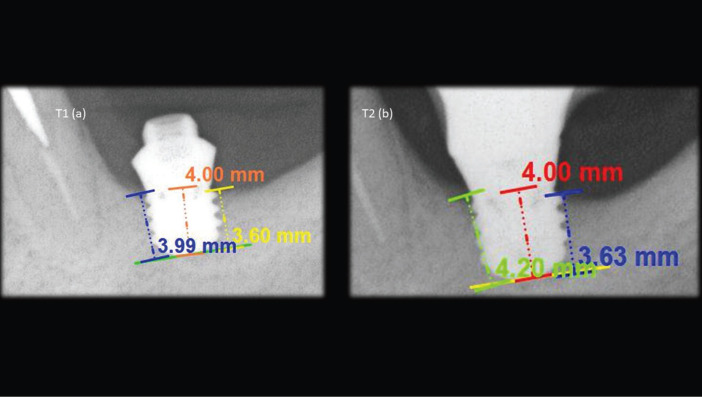
a) measurements made on the radiograph taken immediately after the implant installation; b) measurements made on radiograph after one year of implantation in masticatory function.

After the implants were installed, a periapical radiograph, with parallelism technique, was performed for each implants (T1) and this process was repeated with a minimum period of 1 year in masticatory function (T2), in order to evaluate the marginal bone stability (Fig. 1). The measurements of the mesial and distal bone crestal regions of each implant were selected, from the bone crest to the region parallel to the apex of the implant. The measurements of these crests were based on the original size of the implants for calibration. Analyses were performed independently for the mesial bone crest and distal bone crest (Fig. 2).

For analysis as well as data tabulation, Image J software (National Institutes of Health, Bethesda, Maryland, USA) (13) was used.

Initially, the data were submitted to the D’Agostino & Pearson normality test, which demonstrated their normal distribution. Paired t-test was used to assess for differences in the “bone crest height” variable between T1 and T2. The significance level was set at 5%. Analyses were performed using GraphPad Prism 6.05 software (GraphPad Software, San Diego, California, USA).

After the evaluation of extra-short implants, there was no statistically significant difference between T1 and T2, both in the mesial and distal bone crest (*p*> 0.05) independent of single or united crown rehabilitation (Table 1).

**Table 1:**
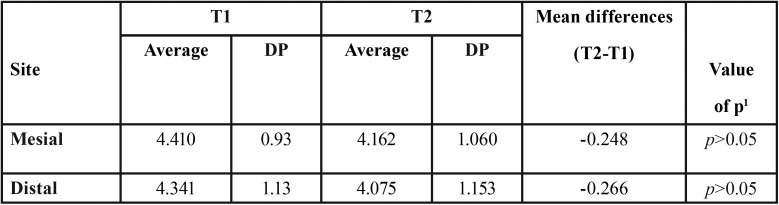
SP: standard deviation.

## Discussion

The use of the longest possible implants has always been an important consideration as it allows for optimal primary stability and greater bone contact area, factors that as consider as key to success (14). However (6) cite in their study that extra-short implants indicated for patients with severe bone resorption, compared to regular implants, provided similar clinical results during 12 months of follow-up. Similar conclusion could be obtained with the present study however using only extra short implants with 4mm.

Low success rate using external fittings and machined surfaces, where 60% of unsuccessful implants were implants classified as short, smaller than 10 mm, and that the success rate of short implants at 6 years were significantly lower than all other long implants (15). This results was similar to (16), reported a success rate of 78.2% for short implants, 7 mm in length, that attributed a low success rate to implant length. The authors did not evaluate the types of connection and implant surfaces.

Today, the low success rate may be related to the type of connection and the type of implant treatment surface (17). Based on these the present study used only implants that treated surface and internal connections (morse taper connection and switching platform).

In a meta-analysis of prospective observational studies (18,11), concluded that the survival of short and extra short implants depends on the crown/implant ratio, implant diameter, implant macrogeometry, surface treatment, and type of connection. The present study with surface-treated implants shows that extra-short implants, which had internal connections and platform switching, achieved good perimplantar bone stability.

Lack of crown implant ratio could induce poor biomechanics (19), resulting in loss of the perimplant bone crest, leading to an early implant loss. However, Anitua (7) in a retrospective study conducted at the Edward Anitua Institute between 2001 and 2009, using 128 extra-short short implants (between 5.5 mm and 8.5 mm) measured the change in the bone crest and concluded that There was no significant relationship between bone loss and implant crown ratio of the short and extra short implants.

A laboratory study used 63 extra-short implants of 5 mm in length and with different diameters: 4.0 mm, 5.0 mm and 6.00 mm, fixed in acrylic resin, placing the platform level of acrylic resin. These implants were loaded with identical cemented prostheses and there was no significant difference in the failure of these implants, all of which were tested to abutment failure or to the maximum load of 900 N. This biomechanics success was also observed in the present study, since all extra short implants in function presented a 2:1 crown / implant disproportion, and there were no significant changes in the perimplantar bone crest level (20).

For best results reducing the risk of perimplantar bone loss, extra-short implants should be single-body (gingival or bone level) with platform switching and surface treatment (21). According to the present study (17) also reported that single-body implants are indicated for the posterior mandible region, where bone quantity is usually limited, thus avoiding a 1.5 to 2mm support loss that would be 25% for an 8mm implant, or 50% in case of 4mm extra short implant used.

Despite the limited sample size and proservation time, extra-short implants are a predictive treatment options for patients with severe bone atrophy in the posterior mandible.

Marginal bone stability observed in the present study, corroborating the biological and biomechanical stability of the extra short implants presented in the literature.

The extra short implants characteristics should be chosen very carefully in order to achieve better predictability.
